# Acute Basophilic Leukemia Arising from Chronic Myeloid Leukemia with Isolated Thrombocytosis

**DOI:** 10.4274/tjh.galenos.2021.2021.0546

**Published:** 2021-12-07

**Authors:** Yun Zhang, Xiaosu Kang, Xiliang Chen, Ting Li

**Affiliations:** 1 The District People’s Hospital of Zhangqiu, Department of Clinical Laboratory, Jinan, China; 2 Shandong College of Traditional Chinese Medicine, Yantai, China; 3 Beijing Ludaopei Hospital, Department of Laboratory and Pathology, Beijing, China

**Keywords:** Acute basophilic leukemia, Chronic myeloid leukemia, Isolated thrombocytosis

## To the Editor,

Acute basophilic leukemia (ABL) is a very uncommon form of acute myeloid leukemia (AML), accounting for <1% of all cases of AML [1]. Most cases have been described as evolving from other hematological diseases, such as chronic myeloid leukemia (CML) and myelodysplastic syndromes [2,3]. CML is one of the classical types of myeloproliferative neoplasms, characterized by the existence of a reciprocal translocation between chromosomes 9 and 22, t(9;22)(q34:q11), resulting in the *BCR-ABL1* fusion gene. Here, we report a very rare case of CML in basophilic blast crisis in a 54-year-old female patient with an 8-year history of CML. It is worth noting that the current World Health Organization (WHO) guidelines would regard this case as CML in the basophilic blast phase rather than ABL.

**Figure 1:**
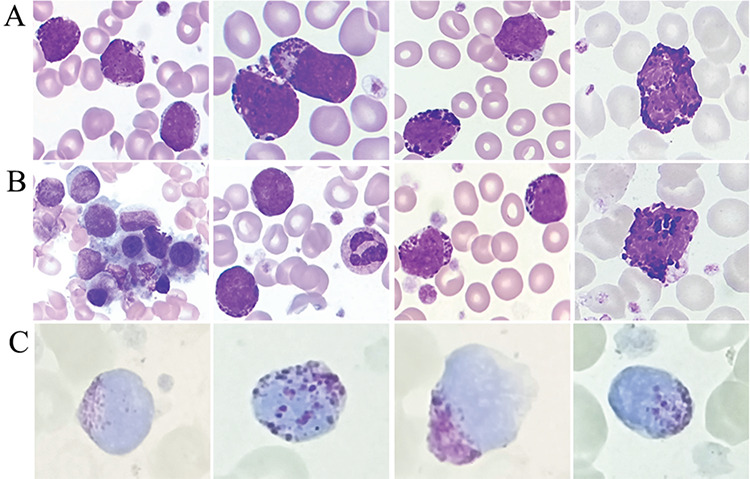
Peripheral blood smear revealed 32% blasts (one-quarter of the blasts containing a variable number of coarse basophilic granules), 2% basophils, and 10% basophilic precursors (A, Wright-Giemsa stain, 1000^x^). Bone marrow aspiration showed hypercellularity with 51.5% blasts (40% of the blasts were metachromatic blasts), 7.5% basophils, and 8% basophilic precursors such as basophilic metamyelocytes and myelocytes; simultaneously, a small amount of dwarf megakaryocytes and numerous agranular blast cells were also observed (B, Wright-Giemsa, 1000x). Basophilic granules in the blasts and basophils exhibited metachromasia with toluidine blue (C).

This 54-year-old female patient had an 8-year history of chronic-phase chronic myeloid leukemia (CML-CP). At the initial diagnosis of CML, a complete blood count showed hemoglobin of 121 g/L and white blood cells of 11.2x10^9^/L, with basophilia and very few immature myeloid cells, accompanied by marked thrombocytosis of 1186x10^9^/L. *BCR-ABL1* (p210 fusion protein) was detected by RT-PCR. Hence, a diagnosis of CML-CP was made. She then started regular oral imatinib at 600 mg/day for 5 years. During that period, she achieved complete remission (CR) several times, and 3 years ago, she had a final bone reexamination that revealed CR with hematological, cytogenetic, and molecular response to imatinib. However, compliance was poor and she discontinued the imatinib treatment. She was subsequently admitted to the hematology department with a low-grade fever for 2 weeks and leukocytosis for 1 day. Physical examination showed no hepatosplenomegaly. Blood tests revealed a total leukocyte count of 32.68x10^9^/L with 3% basophils, absolute basophil count of 0.98x10^9^/L, hemoglobin concentration of 109 g/L, and platelet count of 382x10^9^/L. A peripheral blood (PB) smear revealed 32% blasts (one-quarter of the blasts containing a variable number of coarse basophilic granules), 2% basophils, and 10% basophilic precursors (Figure 1A). Bone marrow (BM) aspiration showed hypercellularity with 51.5% blasts (40% of the blasts were metachromatic blasts), 7.5% basophils, and 8% basophilic precursors such as basophilic metamyelocytes and myelocytes. Simultaneously, a small amount of dwarf megakaryocytes and numerous agranular blast cells were also observed (Figure 1B). Basophilic granules in the blasts and basophils exhibited metachromasia with toluidine blue (Figure 1C). Flow cytometric analysis demonstrated two distinct populations of blastoid cells with one population of CD34+, CD33+, HLA-DR+, CD117+, and CD123+ cells accounting for 31.32%, suggesting immature blast cells, and the second population with 15.99% of indicated blasts showing differentiation to basophils, which were CD33+, CD34+, CD123+, partially CD9+, and partially HLA-DR+. Cytogenetic analysis showed 46,XX,t(9;22)(q34; q11),i(17)(q10)[18]/46,XX,t(9;22)(q34;q11)[2]. Molecular study showed blasts positive for *BCR-ABL* (p210 fusion protein) rearrangement. According to the consensus reports for classification [4], given the history of CML along with the characteristics of dwarf megakaryocytes and t(9;22)(q34;q11) with i(17)(q10), the findings supported the diagnosis of CML in the basophilic blast phase. The patient started two cycles of initial treatment [regimen of idarubicin (60 mg, days 1-3) and cytarabine (0.15 g, days 1-7)] with imatinib (600 mg, by mouth once a day). She then received two cycles of early consolidation chemotherapy of cytarabine (2 g, intravenously, every 12 h, days 1, 3, and 5), two cycles of cytarabine (0.15 g, days 1-7) and homoharringtonine (3 mg, days 1-7), and another two cycles of idarubicin (20 mg, days 1-3) and cytarabine (0.1 g, days 1-7), and she achieved CR. She then began regular oral imatinib at 400 mg/day. To date, she remains in continuous CR.

The term “basophilic leukemia” was first used in 1906 by Joachim. In general, basophilic leukemias should be divided into de novo and secondary forms and acute and chronic variants [4]. ABL was recognized as a distinct entity in the most recent WHO classification of myeloid malignancies [1]. In certain circumstances, the boundary between secondary ABL and BP-CML with increase of basophils is thin; the recently proposed diagnostic criteria for ABL are blasts of ≥20% and immature basophils of ≥40% of nucleated BM or PB cells [4]. Given the clinical history of CML-CP and the percentages and characteristics of blasts and basophils in PB and BM, our case may be considered as CML in basophilic blast crisis. Basophilic blasts are typically characterized by a high N:C ratio, round or irregular nuclei with dispersed chromatin, and moderately basophilic cytoplasm containing a variable number of coarse basophilic granules, which characteristically stain positive in metachromatic staining with toluidine blue. In addition, the blasts are frequently negative for naphthol AS-D chloroacetate esterase (CAE). The lack of CAE reactivity can be helpful in distinguishing blasts of ABL from mast cells [1].

In general, the diagnosis of secondary ABL and CML in basophilic blast crisis is dependent on history, morphology, and molecular profile in combination with immunophenotypic results. However, morphologically, basophilic blasts can be a heterogeneous group varying from agranular features to significantly coarse basophilic presentation. In our case, the majority of the blasts showed no obvious basophilic granules in BM smears. To our knowledge, these two disease entities may have different genetic characteristics. There are many reports of cytogenetic abnormalities in ABL, including the following: t(X;6)(p11.2;q23.3) resulting in *MYBGATA1* [5]; a normal karyotype with *U2AF1* mutation [6]; t(16;21)(p11;q22) generating the *FUS-ERG* fusion gene [7]; t(6;12)(q13;p13.3) with loss of *ETV6* [8]; and loss of *TP53* in the setting of conversion from acute myeloblastic leukemia [9]. Interestingly, Pidala et al. [10] reported secondary ABL from CML with the development of t(7;8)(q32;q13), while our case showed typical t(9;22) translocation and i(17)(q10). The exact significance of the t(7;8) translocation event is not known. Meanwhile, flow cytometric immunophenotyping plays a key role in the definitive diagnosis. Basophilic blasts are usually CD9+, CD25+, CD13+, CD33+, CD123+, CD203c+, and CD11b+, while they are occasionally positive for membrane CD22 and negative for other monocytic markers and CD117 [1]. Additionally, the proposed diagnostic criteria of ABL with blasts of ≥20% and immature basophils of ≥40% of nucleated BM or PB cells are helpful for distinguishing it from CML in basophilic blast crisis [4]. CML in the basophilic blast phase must also be distinguished from de novo ABL and other AML subtypes with basophilia, such as AML with t(6;9)(p23;q34.1), AML with *BCR-ABL1*, and, more rarely, a subtype of lymphoblastic leukemia with prominent coarse granules.

In conclusion, here we have presented an extremely uncommon case of CML in basophilic blast crisis. Due to the rarity and nonspecific morphology of this disease, the combination of clinical history, flow cytometry, and cytogenetic analysis is useful in making a confirmed diagnosis. Since CML in the basophilic blast phase is especially rare, with very few case reports and small collections of cases documented in the literature, more data are needed to ensure the accurate diagnosis and appropriate therapeutic schedule for this unique entity.
